# Synonymous codon-optimized multiplex qPCR for simultaneous detection of *Helicobacter pylori* and precise drug resistance profiling in infected individuals

**DOI:** 10.1128/jcm.01861-25

**Published:** 2026-07-27

**Authors:** Hui-Lan Li, Ya-Li Fan, Tian Zhong, Bi-Yu Lin, Qin-Jin Wang, Jia Xie, Ze-Lin Zhang, Shu-Ping Nie, Hong-Lei Chen, Chao Wu

**Affiliations:** 1 Department of Laboratory Medicine, the Eighth Affiliated Hospital of Sun Yat-sen University 575842, Shenzhen, Guangdong, People's Republic of China; 2 Biological Laboratory of Hetao Cooperation Zone, the Eighth Affiliated Hospital of Sun Yat-sen University 575842, Shenzhen, Guangdong, People's Republic of China; 3 Department of Endoscopy, the Eighth Affiliated Hospital of Sun Yat-sen University 575842, Shenzhen, Guangdong, People's Republic of China; Vanderbilt University Medical Center, Nashville, Tennessee, USA

**Keywords:** *Helicobacter pylori*, antibiotic resistance, multiplex real-time PCR, synonymous codon optimization, *Clarithromycin*, levofloxacin

## Abstract

Antibiotic-resistant *Helicobacter pylori* (*H. pylori*) represents a significant global challenge to the efficacy of eradication therapies, underscoring the urgent need for rapid and precise pretreatment resistance profiling. Traditional genotyping assays frequently neglect synonymous mutations, resulting in false-negative results and reduced diagnostic accuracy. In this study, we developed a single-tube, synonymous codon-optimized multiplex real-time PCR (qPCR) assay for the qualitative detection of *H. pylori* and the simultaneous identification of mutations associated with resistance to clarithromycin (23S rRNA gene) and levofloxacin (*gyrA* gene). The probe design was informed by clinical mutation data and synonymous codon usage patterns from 453 isolates, achieving over 98% coverage of naturally occurring variants. The assay exhibited high sensitivity (limit of detection: 2–20 copies/μL), excellent repeatability (coefficient of variation <5%), and robust specificity. Clinical validation using 316 gastric mucosal specimens demonstrated 97.47% concordance with a commercial infection detection kit and 100% and 98.08% agreement with Sanger sequencing for 23S rRNA and *gyrA* resistance genotyping, respectively. This assay effectively addresses the critical limitation posed by synonymous mutations, providing a rapid, reliable, and clinically accessible tool to guide personalized *H. pylori* eradication therapy and promote antimicrobial stewardship.

**IMPORTANCE:**

Increasing antibiotic resistance in *Helicobacter pylori* has reduced the effectiveness of eradication therapy and highlights the need for accurate resistance profiling before treatment. However, current molecular assays may miss resistant strains because synonymous mutations in probe-binding regions may interfere with detection and produce false-negative results. In this study, we developed a single-tube multiplex real-time PCR assay that incorporates synonymous codon variation into probe design, enabling simultaneous detection of *H. pylori* infection and clarithromycin- and levofloxacin-resistance mutations. By using sequence data from 453 clinical isolates, this assay achieved broad coverage of naturally occurring variants and showed high analytical and clinical performance. This approach addresses an important diagnostic blind spot in *H. pylori* resistance detection and provides a practical tool for pretreatment resistance profiling, individualized therapy selection, and improved antimicrobial stewardship.

## INTRODUCTION

*Helicobacter pylori* (*H. pylori*), a bacterium specifically adapted to the human gastric environment, has infected approximately 50% of the global population (1). It is a principal etiological agent involved in a range of gastroduodenal diseases, including gastritis, peptic ulcer disease, gastric mucosa-associated lymphoid tissue (MALT) B-cell lymphoma, and gastric carcinoma (2). Often characterized as a “silent time bomb” for gastric cancer, the 2024 American College of Gastroenterology (ACG) guidelines recommend a universal “test-and-treat” approach for all infected individuals to mitigate long-term oncogenic risk through prompt bacterial eradication (3).

Nevertheless, the efficacy of standard eradication protocols, particularly clarithromycin-based triple therapy and levofloxacin-containing regimens, has been substantially compromised by the rapid emergence and dissemination of antibiotic resistance (4–10). The widespread resistance of *H. pylori* to key antibiotics poses a significant public health challenge. This challenge is compounded by the high frequency of coresistance, which collectively leads to eradication treatment failure (11–13).

Antibiotic resistance in *H. pylori* is predominantly driven by specific genetic mutations. Clarithromycin resistance is mainly associated with point mutations in the 23S rRNA gene (e.g., A2143G, A2142G, A2142C) (14–16), whereas levofloxacin resistance is primarily conferred by mutations in the quinolone resistance-determining region (QRDR) of the *gyrA* gene, particularly amino acid residues 87 and 91 (17–19). These genetic alterations exhibit a strong correlation with phenotypic resistance in clinical strains (20). Consequently, genotypic detection of these resistance-associated mutations is critical for guiding personalized therapeutic regimens, optimizing treatment selection, improving eradication rates, and limiting the spread of antimicrobial-resistant *H. pylori* strains.

The reliance on genotypic detection is fundamentally constrained by the occurrence of synonymous mutations. Synonymous mutations involve nucleotide substitutions that alter codon sequences without changing the encoded amino acid due to the degeneracy of the genetic code (21, 22). Although historically considered functionally neutral, emerging evidence suggests that synonymous mutations can impact gene expression by affecting mRNA stability, translational efficiency, or splicing (23, 24). More importantly, for molecular diagnostics, synonymous mutations may introduce sequence mismatches within primer or probe binding sites, thereby diminishing hybridization efficiency in PCR- and quantitative real-time PCR (qPCR)-based assays. These mismatches frequently lead to false-negative results, underestimation of resistance prevalence, and compromised accuracy in pretreatment resistance profiling.

Importantly, synonymous mutations within the *H. pylori gyrA* gene have been insufficiently characterized, and most existing studies on *gyrA*-mediated levofloxacin resistance detection have neglected their potential impact on assay performance (25, 26). This oversight likely contributes to the underdetection of resistant strains in clinical settings, resulting in suboptimal therapeutic decisions and unnecessary antibiotic exposure. Therefore, addressing codon degeneracy through targeted assay optimization is essential for achieving comprehensive and reliable *H. pylori* pretreatment resistance screening.

To address this critical gap, we developed a streamlined single-tube multiplex qPCR assay optimized by synonymous codon usage, enabling the detection of *H. pylori* infection and resistance to clarithromycin and levofloxacin simultaneously. By integrating sequencing data from our clinical isolate cohort (*n* = 453) with analyses of synonymous codon usage, the probe design was strategically optimized to ensure comprehensive coverage of naturally occurring variants, mitigating the false negatives from silent mutations that plague conventional assays. This clinically actionable tool enables rapid, precise resistance genotyping, facilitating tailored therapeutic decision-making to improve eradication success and mitigate the transmission of antimicrobial resistance in clinical settings.

## MATERIALS AND METHODS

### Patients and gastric biopsy specimens

This study enrolled 600 patients presenting with gastrointestinal symptoms who underwent upper gastrointestinal endoscopy at the Eighth Affiliated Hospital of Sun Yat-sen University between October 2023 and August 2025. The exclusion criteria included (i) the use of antibiotics, proton pump inhibitors (PPIs), or nonsteroidal anti-inflammatory drugs (NSAIDs) within four weeks prior to endoscopy and (ii) a history of gastric cancer, gastric surgery, or immunosuppressive disorders.

Gastric mucosal biopsies were obtained according to the updated Sydney System (27), with five specimens collected per patient—two from the antrum, two from the corpus, and one from the *incisura angularis*. All biopsies were of adequate size (≥2 mm in diameter) and were processed immediately according to their intended use: two for histopathological examination (hematoxylin and eosin and modified Giemsa staining), one for bacterial culture, and one for clinical qPCR detection of *H. pylori* (DAAN, China), while the remaining sample was snap-frozen at −80°C for subsequent DNA extraction and methodological analyses.

*H. pylori* infection status was defined hierarchically: culture-positive patients were confirmed to be *H. pylori*-positive (isolate cohort, *n* = 453). Among the 147 culture-negative patients, 80 were classified as true negatives on the basis of negative qPCR and histopathology results, and 67 were excluded due to discordant results (any positivity by qPCR or histopathology). For clinical validation of the multiplex qPCR assay, frozen gastric biopsies were obtained from 261 culture-positive and 55 true-negative patients, yielding 316 specimens collected from May 2024 to August 2025. The 261 positive specimens were a subset of the isolate cohort. These 316 patients included 154 males (48.73%) and 162 females (51.27%), with ages ranging from 22–76 years and a median age of 46 years. Endoscopic findings included chronic superficial gastritis (*n* = 188, 59.49%), erosive gastritis (*n* = 57, 18.04%), atrophic gastritis (*n* = 22, 6.96%), gastric ulcers (*n* = 12, 3.80%), and gastric polyps (*n* = 37, 11.71%). Some patients presented with multiple lesions. Patient enrollment and sample selection are illustrated in Fig. S1.

### Clinical *H. pylori* isolates

The isolate cohort comprised 453 culture-positive patients (October 2023–August 2025), with one isolate collected from each patient. The culture procedure was as follows. The biopsy samples were inoculated onto selective agar plates (BLOT, China) and incubated at 37°C for 3–5 days under microaerophilic conditions (5% O_2_, 10% CO_2_, and 85% N_2_). Suspected *H. pylori* colonies were initially identified by Gram staining, which revealed characteristic spiral or curved rods, and confirmed by positive biochemical reactions, including rapid urease, catalase, and oxidase tests. Each batch also included appropriate quality controls: *H. pylori* ATCC 43504 served as the positive control, and *Escherichia coli* ATCC 25922 served as the negative control. For each culture-positive patient, a single colony was selected from the primary culture plate and subcultured for purification. The purified isolate was then propagated and cryopreserved at −80°C in Brucella broth supplemented with 20% glycerol for subsequent analyses.

### Phenotypic antimicrobial susceptibility testing

E-test was used to determine the minimum inhibitory concentrations (MICs) of clarithromycin and levofloxacin in 131 randomly selected clinical isolates of *H. pylori*. Bacterial colonies were suspended in sterile 0.9% NaCl and adjusted to a 3.0 McFarland standard using a DensiChek Plus nephelometer (bioMérieux, France). The suspension was inoculated onto Mueller–Hinton agar supplemented with 5% horse blood. E-test strips (BIO-KONT, China) were placed following the manufacturer’s instructions. The plates were incubated at 37°C under microaerophilic conditions for 3–5 days. The *H. pylori* standard strain ATCC 43504 was used as the quality control strain for the E-test strips. Resistance was interpreted according to the European Committee on Antimicrobial Susceptibility Testing (EUCAST) clinical breakpoints (v16.0), with isolates exhibiting an MIC of >0.25 mg/L for clarithromycin and >1 mg/L for levofloxacin classified as resistant.

### DNA extraction

Genomic DNA was extracted from 453 *H. pylori* isolates using the SteadyPure Bacteria Genomic DNA Extraction Kit (Accurate Biology, China) following the manufacturer’s protocol. For the 316 gastric mucosal tissue samples, approximately 20 mg of tissue was homogenized in sterile saline, and DNA was subsequently extracted using the QIAamp DNA Mini Kit (Qiagen, Germany). DNA concentration and purity were assessed with a NanoDrop 2000 spectrophotometer (Thermo Fisher Scientific, USA), and samples whose A260/A280 ratios were between 1.5 and 2.0 and whose concentrations were ≥10 ng/µL were considered acceptable for further analysis. For the qPCR assay, a fixed volume of 4 μL of extracted DNA was used per reaction, corresponding to a template input mass ranging from 40 to 200 ng. Samples with concentrations below the threshold of 10 ng/μL were subjected to re-extraction. All DNA samples were stored at −20°C until further analysis.

### Drug resistance gene sequencing analysis

Sequencing primers were designed specifically to target key mutation regions in the 23S rRNA and *gyrA* genes associated with antibiotic resistance (Table 1). PCR was performed in a 50 μL reaction mixture containing 25 μL of 2× Super Pfx Master Mix (CWBIO, China), 2.5 μL each of forward and reverse primers (10 μM), approximately 100 ng of genomic DNA, and nuclease-free water to volume. The thermal cycling conditions were as follows: 95°C for 5 min; 40 cycles of 95°C for 20 s, 58°C for 30 s, and 72°C for 45 s; and a final extension at 72°C for 10 min. After verification by gel electrophoresis, the PCR products were sequenced by Shanghai Sangon Biotech Co., Ltd. (China).

**TABLE 1:** Sequences of primers and probes used in this study

Methods	Sequence name	Target	Nucleotide^*a*^	Sequences (5′→3′)
Sanger sequencing	Hp 23S rRNA-F1	*H. pylori* 23S rRNA	/	TACCGACCTGCATGAATGGC
	Hp 23S rRNA-R1			CTCCATAAGAGCCAAAGCCCT
	Hp gyrA-F1	*H. pylori gyrA*	/	ATGAGCGTGATCATAGGGCG
	Hp gyrA-R1			CGGCTTGGTAGAATATCTGGCT
Multiplex qPCR	Hp 23S rRNA-F2	*H. pylori* 23S rRNA	/	GTGAAAATTCCTCCTACCCGC
	Hp 23S rRNA-R2			GCGCATGATATTCCCATTAGCA
	Hp gyrA-F2	*H. pylori gyrA*	/	CGCTAGGATCGTGGGTGATG
	Hp gyrA-R2			CAAAGTTGCCTTGCCCATCC
	Hp 16S rRNA-F	*H. pylori* 16S rRNA	/	GGTGGAGCATGTGGTTTAATTC
	Hp 16S rRNA-R			CAAGCCAGACACTCCACTATT
	hGAPDH-F	*Homo sapiens GAPDH*	/	GATTCCACCCATGGCAAATTC
	hGAPDH-R			CTGGAAGATGGTGATGGGATT
	Hp 23S-A2142G-P	23S rRNA A2142G	GAA	VIC-CAAGACGGGAAGACCCC-BHQ1
	Hp 23S-A2142C-P	23S rRNA A2142C	CAA	VIC-CAAGACGGCAAGACCCC-BHQ1
	Hp 23S-A2143G-P	23S rRNA A2143G	AGA	VIC-AAGACGGAGAGACCCCGT-BHQ1
	Hp gyrA-A260T-P	*gyrA* Ile87	ATT	FAM-CGCAATATCGCCA-MGB
			ATC	FAM-CGCGATATCGCCA-MGB
	Hp gyrA-C261A-P	*gyrA* Lys87	AAA	FAM-CGCTTTATCGCCA-MGB
	Hp gyrA-T261G-P	*gyrA* Lys87	AAG	FAM-CGCCTTATCGCCA-MGB
	Hp gyrA-G271A-P	*gyrA* Asn91	AAT-GCGC	FAM-CGGTTTATAATGCGCTAG-MGB
			AAT-GCAC	FAM-CGGTTTATAATGCACTAG-MGB
			AAT-GCGT	FAM-CGGTTTATAATGCGTTAG-MGB
	Hp gyrA-G271T-P	*gyrA* Tyr91	TAT-GCGC	FAM-CGGTTTATTATGCGCTAG-MGB
			TAT-GCAC	FAM-CGGTTTATTATGCACTAG-MGB
			TAT-GCGT	FAM-CGGTTTATTATGCGTTAG-MGB
	Hp gyrA-A272G-P	*gyrA* Gly91	GGT-GCGC	FAM-CACTAGCGCACCATAAACCGC-BHQ1
			GGT-GCAC	FAM-CACTAGTGCACCATAAACCGC-BHQ1
			GGT-GCGT	FAM-CACTAACGCACCATAAACCGC-BHQ1
	Hp 16S rRNA-P	*H. pylori* 16S rRNA	/	CY5-AGGCTTGACATTGAGAGAATCCGCTAG-BHQ2
	hGAPDH-P	*Homo sapiens GAPDH*	/	ROX-TCCATTGATGACAAGCTTCCCGTTCTC-BHQ2

^*a*^ “/” indicates not applicable; the corresponding primers or probes do not target a specific resistance-associated mutant nucleotide/codon.

The resulting sequences were aligned against the *H. pylori* 26,695 reference genome (NC_000915.1) using SnapGene software (v7.1.2) to identify mutations associated with antibiotic resistance and synonymous codon variations near *gyrA* residues 87 and 91. Control strains harboring confirmed resistance mutations were verified by sequencing (summarized in Table S1) and used as positive controls in the subsequent multiplex qPCR assays. Representative mutant allele sequences have been deposited in the NCBI GenBank database, and the corresponding accession numbers are provided in the Data availability section.

### Primer and probe design

Primers and probes were designed to target four key targets: *H. pylori* 16S rRNA (infection marker), 23S rRNA mutations (A2142C, A2142G, and A2143G; associated with clarithromycin resistance), *gyrA* QRDR mutations (A260T, C261A, T261G, G271A, G271T, and A272G; linked to levofloxacin resistance), and human *GAPDH* (internal control). Key design principles included alignment with the *H. pylori* 26695 reference genome (NC_000915.1) and analysis of clinical mutation data sets (*n* = 453 isolates) to ensure coverage of prevalent variants. Synonymous codon variability at *gyrA* positions 87, 92, and 93, identified through codon usage analysis of clinical isolates, was incorporated to prevent false-negative results caused by silent mutations. Probe specificity was verified via BLAST against nontarget bacterial genomes and the human genome, and primer–probe pairs free of self-complementarity or cross-dimer formation, as predicted by OligoAnalyzer 3.1 software (Integrated DNA Technologies, USA), were selected. The primers and probes used were synthesized by Shanghai Sangon Biotech Co., Ltd. (China), and their sequences are listed in Table 1.

### Establishment of the multiplex qPCR detection system

The optimized multiplex qPCR system, with a total volume of 20 μL, consisted of 1× ChamQ Geno-SNP Probe Master Mix (Vazyme, China), 200 nM of each primer, 100 nM of each probe (except 150 nM for probes Hp 23S A2142G-P and Hp 23S A2143G-P), 4 μL of template DNA, and RNase-free water. Amplification was performed on a QuantStudio 5 Real-Time PCR System (Thermo Fisher, USA) under the following cycling conditions: 95°C for 5 min; 10 cycles of 95°C for 15 s and 61°C for 45 s; and 35 cycles of 95°C for 15 s and 61°C for 45 s, with fluorescence acquisition. For each reaction, nuclease-free water was used as the no-template control (NTC). The wild-type strain ATCC 43504 served as the negative control, and the clinical strain hp132 (genotype: A2143G in 23S rRNA and C261A in *gyrA*) served as the positive control.

### Assay performance evaluation

Serial dilutions of *H. pylori* genomic DNA were prepared to evaluate the linear dynamic range and limit of detection (LOD) of each target in the assay. The dilution series ranged from 2 × 10^5^ to 2 × 10° copies/μL for the 16S rRNA gene; 2 × 10^5^ to 2 × 10^1^ copies/μL for 23S rRNA mutations; and 1 × 10^5^ to 1 × 10^1^ copies/μL for *gyrA* mutations. Each dilution was tested in duplicate, and amplifications with a quantitative cycle (Cq) value ≥26 were considered negative. The LOD was defined as the lowest concentration at which both replicate assays produced a positive amplification signal, meeting all preset Cq and amplification curve criteria.

Precision was evaluated using genomic DNA from the *H. pylori* strain hp132. DNA samples were prepared at concentrations of 10^2^, 10^3^, and 10^4^ copies/μL based on single-copy gene quantification. The intra-assay precision was determined by analyzing 10 replicates per concentration within a single run, and the coefficient of variation (CV) of the Cq values was calculated.

Specificity was evaluated by testing genomic DNA from 11 common gastrointestinal pathogens, including *Staphylococcus aureus*, coagulase-negative *Staphylococcus*, *Pseudomonas aeruginosa*, *Klebsiella pneumoniae*, *E. coli*, *Salmonella* spp., *Enterococcus faecalis*, *Proteus mirabilis*, norovirus, enterovirus 71, and Coxsackievirus A16. The *H. pylori* strain hp132 served as a positive control.

To assess concordance, genomic DNA from the 131 clinical *H. pylori* isolates that underwent phenotypic susceptibility testing was analyzed using multiplex qPCR assay, and the results were compared with those obtained from Sanger sequencing.

### Clinical validation of the multiplex qPCR assay using gastric mucosal specimens

The clinical performance of the multiplex qPCR assay for detecting *H. pylori* infection and associated resistance mutations was rigorously evaluated using 316 gastric mucosal specimens. Detection of *H. pylori* was benchmarked against a commercial nucleic acid test (DAAN, China). Resistance genotyping for 23S rRNA and *gyrA* mutations was validated against Sanger sequencing results, which served as the reference standard.

### qPCR data analysis and validation criteria

The fluorescence data were collected and analyzed using QuantStudio Design and Analysis software (v2.6.0). Cq was defined as the cycle number at which the fluorescence signal surpassed a threshold established within the exponential phase of amplification. The following Cq thresholds were applied for result interpretation: ROX (*GAPDH*, internal control) <22 to confirm sufficient DNA quality and the absence of PCR inhibition; CY5 (16S rRNA) <26 for *H. pylori* detection; and VIC (23S rRNA) or FAM (*gyrA*) <26 for mutation detection, only if the corresponding 16S rRNA signal was positive. A standard curve was deemed acceptable if the difference between each 10-fold dilution corresponded to −3.3 ± 0.3 cycles. Reactions were considered highly efficient and reproducible if the correlation coefficient (*R*²) from all independent experiments ranged between 0.99 and 1.00.

### Statistical analysis

Statistical analyses were performed using SPSS software version 26.0 (IBM Corp., Armonk, NY, USA). Categorical variables are summarized as frequencies and percentages. Comparisons between the multiplex qPCR assay and the reference methods (E-test, commercial qPCR kit, and Sanger sequencing) were performed using 2 × 2 contingency tables. Diagnostic performance metrics, including the concordance rate, sensitivity, specificity, positive predictive value (PPV), and negative predictive value (NPV), were calculated using standard formulas. Agreement between methods was assessed using Cohen’s kappa statistic, with values greater than 0.75 indicating excellent agreement, values between 0.40 and 0.75 indicating moderate agreement, and values below 0.40 indicating poor agreement.

## RESULTS

### Analysis of mutation characteristics in the 23S rRNA and *gyrA* genes of clinical *H. pylori* isolates

**Fig 1:**
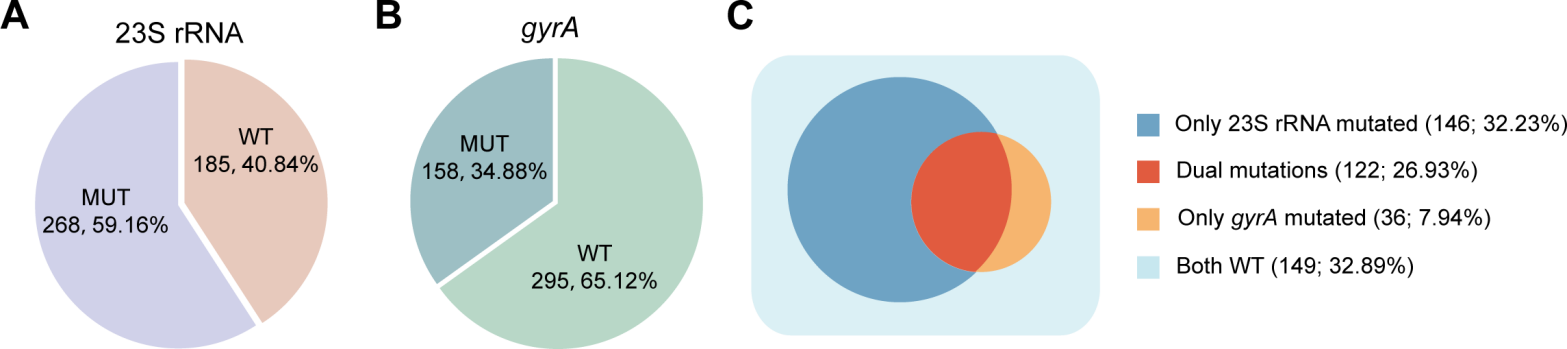
Mutation profiles of the 23S rRNA and *gyrA* genes in 453 clinical *H. pylori* isolates. (**A and B**) Pie charts depicting the proportions of isolates with mutant (MUT) and wild-type (WT) 23S rRNA and *gyrA* genes. (**C**) Venn diagram summarizing the intersection of the mutation profiles between these two genes.

Sanger sequencing was performed on 453 clinical *H. pylori* isolates to investigate mutation profiles, co-occurrence patterns, and key resistance sites in the 23S rRNA and *gyrA* genes. The mutation frequency was significantly higher in the 23S rRNA gene, with 268 isolates (59.16%) exhibiting mutations compared with 185 wild-type isolates (40.84%) (Fig. 1A). In contrast, *gyrA* mutations were less common and were detected in 158 isolates (34.88%) compared with 295 wild-type isolates (65.12%) (Fig. 1B). Co-occurrence analysis revealed 122 isolates (26.93%) harboring concurrent mutations in both genes, indicating multidrug resistance to clarithromycin and levofloxacin (Fig. 1C).

Analysis of specific mutation sites revealed distinct prevalence patterns (Table 2). Among the 268 isolates with 23S rRNA mutations, the A2143G variant was predominant (261, 97.39%), while A2142G (6, 2.24%) and A2142C (1, 0.37%) were rare. Among the 158 isolates carrying *gyrA* mutations, C261A was the most common (58, 36.71%), followed by G271A (42, 26.58%) and A272G (34, 21.52%). The T261G mutation was present in 19 isolates (12.03%), whereas the A260T (8, 5.06%) and G271T (7, 4.43%) variants were relatively rare, collectively representing a minor fraction of the cases.

**TABLE 2:** Mutation profiles of the 23S rRNA and *gyrA* genes in 453 *H. pylori* clinical isolates^*a*^

Gene	Amino acid^*b*^	Genotype	Nucleotide change	*n* (%)
23S rRNA	/	A2142G	AAA > GAA	6 (2.24)
	/	A2142C	AAA > CAA	1 (0.37)
	/	A2143G	AAA > AGA	261 (97.39)
*gyrA*	Ile87	A260T	AAT > ATT	5 (3.16)
			AAC > ATC	3 (1.90)
	Lys87	C261A	AAC > AAA	58 (36.71)
		T261G	AAT > AAG	19 (12.03)
	Asn91	G271A	GAT > AAT	42 (26.58)
	Tyr91	G271T	GAT > TAT	7 (4.43)
	Gly91	A272G	GAT > GGT	34 (21.52)

^*a*^ The apparent discrepancy between the total mutation count and the number of mutant isolates can be accounted for by the presence of isolates harboring concurrent mutations at multiple sites within the *gyrA* gene.

^*b*^ “/” indicates not applicable; 23S rRNA is a non-protein-coding rRNA gene and therefore has no corresponding amino acid residue.

### Analysis of synonymous mutational patterns and codon usage in the *gyrA* gene

**Fig 2:**
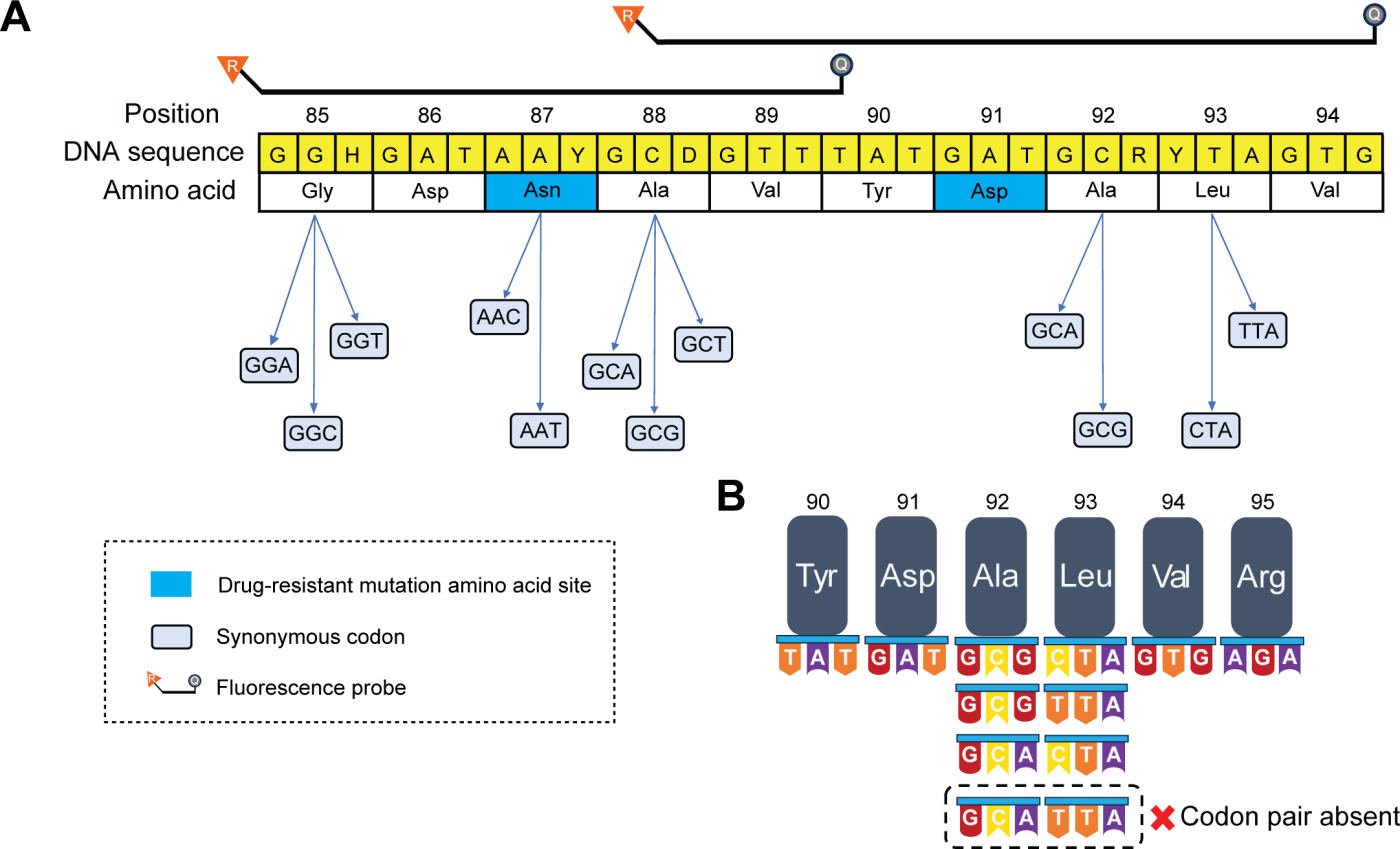
Synonymous codon usage patterns in the *gyrA* gene of *H. pylori*. (**A**) Schematic representation illustrating the variation in synonymous codons at critical amino acid positions 85, 87, 88, 92, and 93. Sites associated with antibiotic resistance (positions 87 and 91) are highlighted in blue. Nucleotide sequences are depicted using degenerate base codes (Y = C/T, D = A/G/T, R = A/G) to capture the observed codon variability. (**B**) Experimentally determined combinations of synonymous codon pairs at adjacent residues 92 (Ala) and 93 (Leu) are presented.

Analysis of *gyrA* sequences from 453 *H. pylori* isolates revealed multiple synonymous mutations near key quinolone-resistance sites at amino acid residues 87 and 91. These synonymous substitutions were identified at positions 85, 87, 88, 92, and 93, each exhibiting two to three different synonymous codons (Fig. 2A). Codon usage analysis demonstrated distinct synonymous preferences among clinical isolates (Table 3). At position 87, the codons AAC (52.89%) and AAT (47.11%) were used comparably. A pronounced bias was observed at position 92 (GCG: 77.65% vs GCA: 22.35%) and position 93 (CTA: 85.40% vs TTA: 14.60%). Codon usage was highly conserved at position 85 (GGC: 99.34%) and position 88 (GCG: 98.45%). At position 91, aspartic acid was exclusively encoded by the GAT codon. The synonymous GAC codon was not detected in any of the 453 isolates.

**TABLE 3:** Frequencies of synonymous codon usage in amino acid residues adjacent to *gyrA* mutations^*a*^

Amino acid	Degenerate codon	*n* (%)
Gly85	GGC	450 (99.34)
	GGA	1 (0.22)
	GGT	2 (0.44)
Asn87	AAC	201 (52.89)
	AAT	179 (47.11)
Ala88	GCG	444 (98.45)
	GCA	5 (1.11)
	GCT	2 (0.44)
Ala92	GCG	351 (77.65)
	GCA	101 (22.35)
Leu93	CTA	386 (85.40)
	TTA	66 (14.60)

^*a*^ The total number of synonymous codon instances at some position is less than 453 because isolates with missense mutations were excluded from the wild-type codon analysis.

Further analysis of codon pairing at adjacent amino acid positions revealed nonrandom associations, indicating preferential codon combinations. For example, although four theoretical combinations exist between codons at positions 92 (GCG/GCA) and 93 (CTA/TTA), “GCA-TTA” pairing was not detected in any of the clinical isolates examined (Fig. 2B), suggesting its rarity or absence in natural populations.

### Development of the multiplex qPCR assay for detecting drug-resistance mutations in *H. pylori*

The multiplex qPCR assay was designed to detect key resistance-associated mutations in the 23S rRNA gene (A2142C, A2142G, and A2143G) and the *gyrA* gene (A260T, C261A, T261G, G271A, G271T, and A272G). Additional targets included the *H. pylori* 16S rRNA gene for infection confirmation and the human *GAPDH* gene as an internal control. To maximize detection coverage, *gyrA* probes were designed by incorporating the most prevalent synonymous codons at amino acid positions 87, 92, and 93, based on prior codon usage analysis (Table 3). Customized multiprobe panels were constructed to address mutations exhibiting substantial codon degeneracy, resulting in two probes for A260T and three probes each for G271A, G271T, and A272G. Conversely, conventional single probes were employed for C261A and T261G mutations. This comprehensive approach yielded detection coverage exceeding 98% for naturally occurring *H. pylori* variants (Table 3). Systematic optimization of the annealing temperature, primer concentrations, and probe concentrations ensured specific and efficient amplification of all the targets (original data available upon request).

### Analytical validation of the multiplex qPCR assay

**Fig 3:**
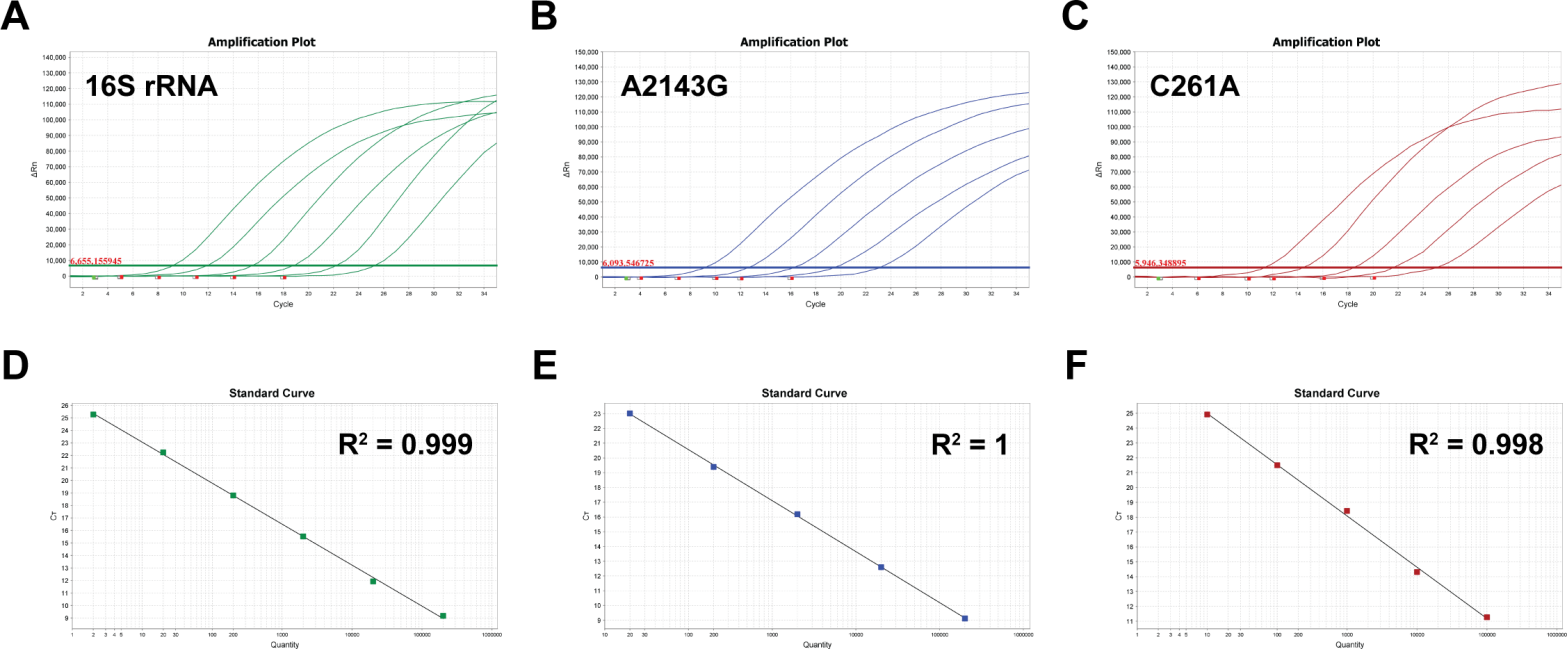
Analytical sensitivity and linearity of the multiplex qPCR assay. Amplification profiles and the corresponding standard curves were generated from serial dilutions of *H. pylori* genomic DNA for (**A and D**) the 16S rRNA gene (2 × 10^5^–2 × 10° copies/μL; *R*^2^ = 0.999), (**B and E**) the 23S rRNA A2143G mutation (2 × 10^5^–2 × 10^1^ copies/μL; *R*^2^ = 1.000), and (**C and F**) the *gyrA* C261A mutation (1 × 10^5^–1 × 10^1^ copies/μL; *R*^2^ = 0.998).

The assay demonstrated high sensitivity, with LODs of 2 copies/μL for the 16S rRNA gene, 20 copies/μL for 23S rRNA mutations, and 10 copies/μL for *gyrA* mutations. The linear dynamic ranges spanned six orders of magnitude for the 16S rRNA gene (2 × 10^5^ to 2 × 10^0^ copies/μL; *R*^2^ = 0.999) and five orders of magnitude for the 23S rRNA mutations (2 × 10^5^ to 2 × 10^1^ copies/μL; *R*^2^ = 0.993–1.000) and *gyrA* mutations (1 × 10^5^ to 1 × 10^1^ copies/μL; *R*^2^ = 0.994–0.999). Representative linearity data for the predominant mutations A2143G (23S rRNA) and C261A (*gyrA*) are shown in Fig. 3.

The assay showed excellent repeatability, with CVs of the Cq values less than 5% across 10 replicates at 10^2^, 10^3^, and 10^4^ copies/μL (Table S2). Specificity was confirmed by testing 11 common gastrointestinal pathogens in the assay, and no cross-reactivity was observed (Fig. S2). Clinical concordance was evaluated using 131 *H. pylori* isolates, revealing strong agreement with the Sanger sequencing results (Table S3). For the 23S rRNA gene, the assay achieved 99.24% concordance, with 100% sensitivity, 97.56% specificity, and a kappa coefficient of 0.982 (95% CI: 0.947–1.000). Complete concordance was observed for *gyrA* mutations, with all 49 mutant and 82 wild-type isolates correctly classified. To further substantiate the clinical utility of the assay, we compared the genotypic resistance profiles obtained by the multiplex qPCR assay with those obtained by phenotypic susceptibility testing using the E-test method on the same 131 *H. pylori* isolates (Table S4). With respect to resistance to clarithromycin, the results of the qPCR assay demonstrated 98.47% (129/131) concordance with those of the phenotypic resistance test, with two isolates carrying 23S rRNA mutations by qPCR but displaying phenotypic susceptibility. For levofloxacin resistance, the overall concordance rate was 96.95% (127/131), with four isolates showing phenotypic resistance despite negative qPCR results for *gyrA* mutations. These findings confirm the high reliability and accuracy of the developed qPCR assay for the clinical detection of antibiotic-resistant *H. pylori*.

### Clinical application of the multiplex qPCR assay in gastric mucosal specimens

To evaluate the performance of the multiplex qPCR assay in complex clinical settings, it was applied to 316 gastric mucosal specimens. Compared with a commercial reference kit for *H. pylori* screening, the assay demonstrated an overall concordance of 97.47% (Table 4), with a sensitivity of 98.85%, a specificity of 98.18%, and a kappa coefficient of 0.957 (95% CI: 0.845–0.966), confirming its high reliability for clinical detection.

**TABLE 4:** Comparison of the developed multiplex qPCR assay with commercial *H. pylori* nucleic acid detection method^*a*^

This study	Commercial qPCR kit		Accordance rate (%)	Se (%)	Sp (%)	PPV (%)	NPV (%)	κ (95%CI)
	Positive	Negative						
Positive	258	1	98.73	98.85	98.18	99.62	94.74	0.957 (0.914–0.999)
Negative	3	54						

^*a*^ Sp, specificity; Se, sensitivity; PPV, positive predictive value; NPV, negative predictive value; κ, Cohen’s kappa coefficient.

Among 316 clinical specimens, high-quality Sanger sequencing data for both target genes were obtained from 261 samples (Table 5). With respect to the 23S rRNA gene, the multiplex qPCR results demonstrated 100% concordance with the sequencing results, identifying 149 samples with mutations and 112 wild-type samples. For the *gyrA* gene, the assay achieved 100% specificity (166/166 wild-type samples correctly identified) and 95.79% sensitivity (91/95 mutation-positive samples detected), resulting in an overall concordance rate of 98.08%. The four *gyrA* mutation-positive samples that were not detected harbored a rare synonymous variant (GT13, GT126, or GT190) or a dual missense mutation at residues 87–88 (GT282), which were not targeted by the current probe design (Fig. S3).

**TABLE 5:** Performance comparison of the developed multiplex qPCR assay and sequencing for detecting *H. pylori* clarithromycin and levofloxacin resistance mutations in gastric mucosal biopsies^*a*^

Gene	This study	Sanger sequencing		Accordance rate (%)	Se (%)	Sp (%)	PPV (%)	NPV (%)	κ (95%CI)
		Mutation	Wild-type						
23S rRNA	Positive	149	0	100.00	100.00	100.00	100.00	100.00	1.000 (1.000–1.000)
	Negative	0	112						
*gyrA*	Positive	91	0	98.47	95.79	100.00	100.00	97.65	0.967 (0.934–0.999)
	Negative	4	166						

^*a*^ Sp, specificity; Se, sensitivity; PPV, positive predictive value; NPV, negative predictive value; κ, Cohen’s kappa coefficient.

## DISCUSSION

The global challenge of treating antibiotic-resistant *H. pylori* has transformed clinical practice, shifting from empiric treatment to individualized therapeutic strategies guided by pretreatment resistance profiling. This change arises from the understanding that antibiotic resistance constitutes not only a biological challenge but also a major barrier to effective disease management. The present study addresses a persistent gap by developing a synonymous codon-optimized multiplex quantitative PCR assay. This methodology aims to overcome the inherent limitations of conventional genotypic detection methods, thereby meeting current clinical and epidemiological needs.

Historically, the degeneracy of the genetic code has been regarded as a neutral characteristic, with synonymous mutations considered functionally inconsequential. However, recent advances in microbial genetics have challenged this assumption, demonstrating that synonymous variations can affect gene expression, protein folding, and even bacterial fitness (28, 29). Neglecting the potential impact of synonymous codon variations in molecular diagnostics can lead to sequence mismatches between primers/probes and target templates, thereby compromising assay sensitivity.

In *H. pylori*, this issue is exacerbated by its high genetic plasticity, driven by natural transformation and selective pressures from antibiotics and host immunity (30, 31). Our analysis of clinical isolates confirms the frequent occurrence of synonymous mutations within the *gyrA* gene, particularly near key resistance-associated residues at positions 87 and 91. These variations are nonrandom (32, 33). Codon usage at positions 87, 92, and 93 exhibits distinct preferences, reflecting evolutionary selection for translational efficiency (34) or GyrA structural stability. The absence of the “GCA-TTA” codon pair at positions 92–93 further supports the notion that synonymous variation is subject to functional constraints rather than random drift (35–37).

Several multiplex qPCR assays have been reported for detecting *H. pylori* resistance, targeting canonical mutations in 23S rRNA and *gyrA* (25, 26, 38). However, these assays are often designed on the basis of reference genomes or limited data sets and overlook the potential of synonymous codon variations to impair probe binding—a key limitation that may lead to false-negative results with strains carrying silent mutations within probe regions. Our assay addresses this gap through a data-driven, synonymous codon optimization strategy. By analyzing codon usage in 453 clinical isolates, we designed multiprobe panels that achieved over 98% coverage of naturally occurring variants, outperforming conventional single-probe designs. For instance, while prior studies typically use a single probe for *gyrA* codon 91 mutations (25), our approach employs three probes to account for codon degeneracy at positions 92–93, ensuring robust detection across genetically diverse strains. This data-driven probe design distinguishes our work from studies that have focused exclusively on technical validation while facilitating increased alignment with real-world clinical genetic diversity.

Furthermore, the codon-informed design strategy developed in this study has broader applicability beyond *H. pylori* and could serve as a general framework for improving molecular assays targeting other pathogens with high frequencies of synonymous mutations. While this broader applicability requires future validation, our study advances the field by both improving resistance mutation coverage for *H. pylori* and establishing a generalizable, biologically grounded framework for microbial diagnostic design.

E-test phenotypic validation on 131 clinical isolates confirmed the robustness of our genotypic approach, demonstrating high concordance rates for both clarithromycin and levofloxacin. These findings validate that our synonymous codon-optimized probe design effectively captures the vast majority of clinically relevant resistance variants. The infrequent discordant cases may be attributable to mixed infections, borderline MIC values, or alternative resistance mechanisms (e.g., *gyrB* mutations or efflux pumps) not covered by the assay. Such sporadic discrepancies do not compromise its overall clinical utility as a reliable tool for guiding personalized *H. pylori* infection treatment.

Multiplex qPCR has become a cornerstone of rapid molecular diagnostics, enabling the simultaneous detection of multiple targets within a single reaction. The single-tube format streamlines laboratory workflows and reduces the risk of contamination, which is critical in high-throughput clinical settings. Compared with nested PCR approaches, this method minimizes human error and shortens the turnaround time to under 3 h. This facilitates timely therapeutic decision-making, which can ultimately improve patient compliance and clinical outcomes. Furthermore, its compatibility with standard real-time PCR platforms eliminates the need for specialized instrumentation, increasing accessibility in resource-limited environments where next-generation sequencing is unavailable.

While the primary objective of *H. pylori* resistance testing is to improve eradication rates, its implications extend to antimicrobial stewardship. The overuse and misuse of antibiotics drive the dissemination of resistance. Pretreatment resistance profiling helps clinicians avoid ineffective prescriptions. This approach reduces the selective pressure that favors the emergence and spread of multidrug-resistant (MDR) strains. Our finding that 26.93% of clinical isolates harbor comutations in 23S rRNA and *gyrA* underscores the urgency of this issue. MDR *H. pylori* infection is associated with significantly reduced eradication rates, even with salvage therapies, and its prevalence is increasing globally, particularly in regions with high antibiotic consumption. The ability of the assay to simultaneously detect clarithromycin and levofloxacin resistance provides a comprehensive resistance profile, facilitating the selection of appropriate combination therapies. This capability is especially critical in settings with limited second-line treatment options.

Beyond clinical diagnostics, the assay holds significant potential for use in epidemiological surveillance. Rapid detection of resistance mutations in clinical isolates enables monitoring of trends in prevalence over time and across regions, thereby informing local treatment guidelines. The Maastricht V/Florence Consensus Report and the 2017 ACG clinical guidelines recommend against the use of clarithromycin-based triple therapy in areas where the prevalence of clarithromycin resistance exceeds 15% (39, 40). Our assay provides a cost-effective and scalable tool for generating such data, supporting evidence-based public health policies. Clinical guidelines recommend resistance testing for all infected individuals, especially those in high-prevalence regions (41). However, widespread implementation has been hindered by the limited accessibility of diagnostic tools and their inadequate detection coverage. Our assay overcomes these barriers, facilitating the adoption of “test-and-treat” strategies aimed at reducing the population burden of *H. pylori*-associated diseases.

Notably, this assay is designed for the qualitative detection of hotspot mutations. While this approach does not distinguish between different mutation subtypes, confirm the coexistence of multiple mutations within the same gene, or differentiate between single-strain infections and mixed bacterial populations, it is clinically oriented. The presence of any resistance-associated mutation in the 23S rRNA or *gyrA* gene—regardless of whether it originates from a single strain or a subpopulation within a mixed infection—is sufficient to predict treatment failure and guide therapeutic decisions. According to the Maastricht VI/Florence Consensus Report (42), the detection of a resistant subpopulation in a clinical sample contraindicates the use of the corresponding antibiotic. Therefore, detailed subtyping or discrimination of infection type is unnecessary for first-line clinical management. Sequencing remains a valuable complementary tool for comprehensive genotyping and epidemiological surveillance.

### Limitations

Despite these advances, several limitations should be acknowledged. First, the functional significance of synonymous mutations in *H. pylori* warrants further investigation. While our assay accounts for these mutations to increase detection accuracy, their biological roles remain to be elucidated. In addition, this assay is optimized for common mutations and synonymous variants identified in our local clinical isolates. It does not cover rare resistance-associated mutations or synonymous variants or other resistance determinants outside the QRDR. Future optimization could incorporate advanced probe designs (e.g., blocker probes or multicolor melting curve analysis) or integrate whole-genome sequencing as a complementary tool for detecting rare variants. Finally, expanding the assay to include other clinically relevant resistance determinants (e.g., metronidazole, amoxicillin, and tetracycline) and validating it through multicenter studies across diverse geographic populations will be critical to increasing its generalizability and clinical utility.

In conclusion, this study addresses the challenge of false-negative results caused by synonymous mutations in *H. pylori* resistance detection through the development of a synonymous codon-optimized, single-tube multiplex qPCR assay. This assay can simultaneously identify *H. pylori* infection and resistance to clarithromycin and levofloxacin. Validated both analytically and clinically, the assay exhibits high sensitivity and specificity, covers more than 98% of the relevant variants, and demonstrates excellent agreement with reference standards. This robust diagnostic tool supports personalized eradication therapy and contributes to antimicrobial stewardship efforts.

## Data Availability

The source data and reagents can be obtained from the corresponding author upon reasonable request. The representative nucleotide sequences of mutant alleles for the 23S rRNA gene and the *gyrA* gene generated in this study have been deposited in GenBank under accession numbers PZ317604 to PZ317606 for 23S rRNA and PZ324866 to PZ324878 for *gyrA*.

## SUPPLEMENTAL MATERIAL

Supplemental figures and tables jcm.01861-25-s0001.docx Figures S1 to S3 and Tables S1 to S4.

ASM does not own the copyrights to Supplemental Material that may be linked to, or accessed through, an article. The authors have granted ASM a non-exclusive, world-wide license to publish the Supplemental Material files. Please contact the corresponding author directly for reuse.
