# Supplementary material for: Synonymous codon-optimized multiplex qPCR for simultaneous detection of Helicobacter pylori and precise drug resistance profiling in infected individuals
Source: J Clin Microbiol. 2026 Jul 27;64(8):e01861-25. doi: 10.1128/jcm.01861-25 (PMC13463915; doi:10.1128/jcm.01861-25)
Supplement: Supplementary file 1 [file jcm.01861-25-s0001.docx]

**Synonymous Codon-Optimized Multiplex qPCR for Simultaneous Detection of *Helicobacter pylori* and Precise Drug Resistance Profiling in Infected Individuals**

Hui-Lan Li**^#^**^1, 2^, Ya-Li Fan**^#^**^1, 2^, Tian Zhong^1, 2^, Bi-Yu Lin^1, 2^, Qin-Jin Wang^1, 2^, Jia Xie^1, 2^, Ze-Lin Zhang^1, 2^, Shu-Ping Nie*^1, 2^, Hong-Lei Chen*^2, 3^, Chao Wu*^1, 2^

^1^ Department of Laboratory Medicine, the Eighth Affiliated Hospital of Sun Yat-sen University, Shenzhen, Guangdong 518033, P.R. China.

^2^ Biological Laboratory of Hetao Cooperation Zone, the Eighth Affiliated Hospital of Sun Yat-sen University, Shenzhen, Guangdong 518033, P.R. China.

^3^ Department of Endoscopy, the Eighth Affiliated Hospital of Sun Yat-sen University, Shenzhen, Guangdong 518033, P.R. China.

**^#^**Hui-Lan Li and Ya-Li Fan contributed equally to this work and share first authorship.

Hui-Lan Li: lihlan3@163.com

Ya-Li Fan: fanyalilili@163.com

Tian Zhong: ztzt622@163.com

Bi-Yu Lin: beryl_lam@qq.com

Qin-Jin Wang: wqinjin@163.com

Jia Xie: xiejia1996@126.com

Ze-Lin Zhang: 351211146@qq.com

Shu-Ping Nie: nieping2@126.com

Hong-Lei Chen: chenhlei3@mail.sysu.edu.cn

Chao Wu: wuch57@mail.sysu.edu.cn

***Correspondence:**

**Chao Wu:** Department of Laboratory Medicine, The Eighth Affiliated Hospital of Sun Yat-sen University, 3025 Shennan Middle Road, Shenzhen 518033, P.R. China.

Email: wuch57@mail.sysu.edu.cn; chaowu261@163.com

**Hong-Lei Chen**: Department of Endoscopy, The Eighth Affiliated Hospital of Sun Yat-sen University, 3025 Shennan Middle Road, Shenzhen 518033, P.R. China.

Email: chenhlei3@mail.sysu.edu.cn

**Shu-Ping Nie**: Department of Laboratory Medicine, The Eighth Affiliated Hospital of Sun Yat-sen University, 3025 Shennan Middle Road, Shenzhen 518033, P.R. China.

Email: nieping2@126.com

**Supplementary Figures**

**Fig. S1. Flowchart of patient enrollment and sample selection.**

**Fig. S2.** **Specificity evaluation of the multiplex qPCR assay.** Amplification curves generated from 11 non-target gastrointestinal pathogens show no specific amplification (i.e., no cross-reactivity) across the three detection channels: **(A)** *H. pylori* identification (Channel hp, 16S rRNA), **(B)** clarithromycin resistance (Channel CLR, 23S rRNA), and **(C)** levofloxacin resistance (Channel LVX, *gyrA*). *H. pylori* strain hp132 was included as a positive control (PC).

**Fig. S3. Verification of *gyrA* mutations in qPCR-discordant specimens. Sanger sequencing chromatograms identify probe-binding region variants in four discrepant cases: (A–C) synonymous mutations in GT13 (88-GCA), GT126 (85-GGA), and GT190 (85-GGT); (D) a nonsynonymous mutation in GT282 (88-CCG, Ala88Pro). The relevant bases are demarcated by dashed boxes (red: synonymous; orange: nonsynonymous).**

**Supplementary Tables**

**Table S1.** **Genotypes of positive control strains for 23S rRNA and *gyrA* genes**

| Strain ID | Target gene | Mutation type | Nucleotide change | Amino acid change | 92–93 codon pair |
| --- | --- | --- | --- | --- | --- |
| hp127 | 23S rRNA | A2142G | AAA>GAA | / | / |
| hp245 | 23S rRNA | A2142C | AAA>CAA | / | / |
| hp99 | 23S rRNA | A2143G | AAA>AGA | / | / |
| hp50 | *gyrA* | A260T | AAT>ATT | Ile87 | / |
| hp322 | *gyrA* | A260T | AAC>ATC | Ile87 | / |
| hp132 | *gyrA* | C261A | AAC>AAA | Lys87 | / |
| hp71 | *gyrA* | T261G | AAT>AAG | Lys87 | / |
| hp94 | *gyrA* | G271A | GAT>AAT | Asn91 | GCG-CTA |
| hp120 | *gyrA* | G271A | GAT>AAT | Asn91 | GCA-CTA |
| hp106 | *gyrA* | G271A | GAT>AAT | Asn91 | GCG-TTA |
| hp115 | *gyrA* | G271T | GAT>TAT | Tyr91 | GCG-CTA |
| hp311 | *gyrA* | G271T | GAT>TAT | Tyr91 | GCA-CTA |
| hp410 | *gyrA* | G271T | GAT>TAT | Tyr91 | GCG-TTA |
| hp73 | *gyrA* | A272G | GAT>GGT | Gly91 | GCG-CTA |
| hp95 | *gyrA* | A272G | GAT>GGT | Gly91 | GCA-CTA |
| hp92 | *gyrA* | A272G | GAT>GGT | Gly91 | GCG-TTA |

**Table S2. Precision assessment of the multiplex qPCR assay**

|  | 10^2^ copies/μL | | | 10^3^ copies/μL | | | 10^4^ copies/μL | | | |
| --- | --- | --- | --- | --- | --- | --- | --- | --- | --- | --- |
| Channel | hp^a^ | CLR^b^ | LVX^c^ | hp | CLR | LVX | hp | CLR | LVX |  |
| 1 | 17.790 | 18.060 | 20.574 | 14.416 | 14.827 | 17.113 | 10.717 | 11.313 | 12.515 |  |
| 2 | 17.876 | 17.734 | 19.395 | 14.210 | 14.634 | 16.639 | 11.143 | 11.954 | 13.589 |  |
| 3 | 17.494 | 17.366 | 19.394 | 14.148 | 14.388 | 15.775 | 10.997 | 11.321 | 13.058 |  |
| 4 | 17.520 | 17.586 | 19.669 | 14.830 | 14.881 | 16.588 | 11.565 | 11.584 | 12.882 |  |
| 5 | 17.287 | 17.549 | 19.240 | 14.646 | 14.649 | 15.206 | 11.218 | 11.280 | 12.688 |  |
| 6 | 17.410 | 17.283 | 18.941 | 14.289 | 14.388 | 15.989 | 11.007 | 11.315 | 12.840 |  |
| 7 | 17.596 | 17.830 | 19.787 | 14.463 | 14.331 | 15.807 | 11.361 | 11.340 | 13.197 |  |
| 8 | 17.602 | 17.673 | 19.613 | 14.475 | 14.158 | 16.441 | 10.933 | 11.238 | 12.050 |  |
| 9 | 17.440 | 17.547 | 18.819 | 14.395 | 14.401 | 16.461 | 10.805 | 11.173 | 12.581 |  |
| 10 | 17.402 | 17.256 | 20.069 | 13.937 | 13.738 | 15.363 | 10.841 | 11.691 | 12.999 |  |
| CV | 1.03% | 1.43% | 2.66% | 1.76% | 2.33% | 3.77% | 2.39% | 2.14% | 3.27% |  |

Notes: ^a^*H. pylori* detection, ^b^Clarithromycin resistance detection (23S rRNA), ^c^Levofloxacin resistance detection (*gyrA*).

**Table S3.** **Comparison of multiplex qPCR and sanger sequencing for genotyping 131 *H. pylori* isolates**

| Gene | This study | Sanger Sequencing | | Accordance rate (%) | Se (%) | Sp (%) | PPV (%) | NPV (%) | κ (95%CI) |
| --- | --- | --- | --- | --- | --- | --- | --- | --- | --- |
|  |  | Mutation | Wild-type |  |  |  |  |  |  |
| 23S rRNA | Positive | 90 | 1 | 99.24 | 100.00 | 97.56 | 98.90 | 100.00 | 0.982 (0.947-1.000) |
|  | Negative | 0 | 40 |  |  |  |  |  |  |
| *gyrA* | Positive | 49 | 0 | 100.00 | 100.00 | 100.00 | 100.00 | 100.00 | 1.000 (0.947-1.000) |
|  | Negative | 0 | 82 |  |  |  |  |  |  |

Notes: Sp, specificity; Se, sensitivity; PPV, positive predictive value; NPV, negative predictive value; κ, Cohen’s kappa coefficient.

**Table S4. Concordance between the multiplex qPCR and phenotypic susceptibility testing for detecting clarithromycin and levofloxacin resistance in *H. pylori* isolates**

| This study | Phenotype | | Accordance rate (%) | Se (%) | Sp (%) | PPV (%) | NPV (%) | κ (95%CI) |
| --- | --- | --- | --- | --- | --- | --- | --- | --- |
|  | R | S |  |  |  |  |  |  |
| Clarithromycin |  |  |  |  |  |  |  |  |
| Positive | 88 | 2 | 98.47 | 100.00 | 95.35 | 97.78 | 100.00 | 0.965 (0.917-1.000) |
| Negative | 0 | 41 |  |  |  |  |  |  |
| Levofloxacin |  |  |  |  |  |  |  |  |
| Positive | 49 | 0 | 96.95 | 92.45 | 100.00 | 100.00 | 95.12 | 0.936 (0.874-0.998) |
| Negative | 4 | 78 |  |  |  |  |  |  |

Notes: Sp, specificity; Se, sensitivity; PPV, positive predictive value; NPV, negative predictive value; κ, Cohen’s kappa coefficient; R, resistant; S, susceptible.
